# University Students' Understanding and Utilization of Food Labels: A Cross-Sectional Study

**DOI:** 10.1155/ijfo/7391826

**Published:** 2025-02-26

**Authors:** Sharifa AlBlooshi, Linda Smail, Asya Aldayyani, Falak Zeb, Alia Ibrahim

**Affiliations:** ^1^Department of Health Sciences, College of Natural and Health Sciences, Zayed University, Dubai, UAE; ^2^Department of Computational Systems, College of Interdisciplinary Studies, Zayed University, Dubai, UAE; ^3^Department of Health Sciences, College of Natural and Health Sciences, Zayed University, Abu Dhabi, UAE; ^4^Research Institute of Medical and Health Sciences, University of Sharjah, Sharjah, UAE

**Keywords:** attitudes, food labeling, health knowledge, practice, understanding, utilization

## Abstract

**Introduction:** This study is aimed at investigating Zayed University students' knowledge, attitudes, and practices (KAPs) regarding food labels and at identifying key predictors of food label use through logistic regression analysis.

**Methods:** This quantitative cross-sectional study used a validated questionnaire to elicit data on KAPs regarding the use of nutritional information and food labels. This study was conducted from January to May 2023 in the UAE among students from Zayed University. A total of 1153 students aged 18 and above from Zayed University participated in the study. They were recruited using snowball sampling. Descriptive statistics were obtained using the Statistical Package for the Social Sciences (SPSS) version 26.

**Results:** We found a positive level of knowledge regarding food labeling (89.9%). Over half the participants viewed food labels positively as around 55.6% reported checking labels and 58.6% replaced food based upon labels while 67.0% checked calories, and 49.7% checked nutritional value. Approximately 60% made their choices based on cost. Over 80% reported checking expiry dates and avoiding expired items. Marital and employment status was the only variables to influence attitudes toward food labeling and checking labels.

**Conclusion:** Our findings show that there is great potential for education regarding food labels to be effective in improving dietary practices.

## 1. Introduction

Unhealthy diets have long been proven to contribute to the development of noncommunicable diseases. As a result, the public and researchers have become interested in learning more about food labels to improve their diets and avoid such diseases [1–3]. A food label is a small table on the back of a food product that contains the product's nutritional information, such as fat, protein, carbohydrates, and micronutrients per serving size. In addition to the percentage of daily value, this food label fulfills each nutrient based on a 2000-cal diet [4, 5]. This information can significantly influence consumer behavior in terms of their dietary choices. When used correctly, it is an educational tool allowing the public to make informed decisions about their dietary practices. For instance, it can guide people who always choose products high in calories, salt, and fat to select less calorie-dense products, those containing healthy fats, and those that have a lower salt content [1, 2, 6–8].

However, what do people look for in these labels? Studies found that the most common factors people consider when choosing food products include price, expiry date, taste, and brand [9–11]. Likewise, a study done in Al Ain in the United Arab Emirates found that approximately 80% of consumers only pay attention to product validity and expiration dates [12]. On the other hand, people on specific diets tend to search for the number of calories, food allergies, ingredients, and health claims [13]. Those more concerned with their health tend to have a more positive attitude regarding reading food labels when purchasing products [14]. This is because they are typically interested in whether products are low in sugar, fat, and calories to meet their goal of a healthy lifestyle and a balanced weight [15].

However, reading labels does not always correlate with choosing healthier food [16]. People may sometimes misuse food labels. For example, people may only look at certain parts or health claims on the label while disregarding other aspects, such as focusing on sugar, calories, or the latest health trend [17]. This pattern can also be present among people with certain diseases. Someone with high blood pressure may shop for products low in sodium while ignoring that they might be high in trans fat or sugar [9]. Furthermore, studies show that people may look only at the calories when choosing foods with the goal of weight loss [1]. Another common mistake is that people may misunderstand the serving size and overconsume [18]. In some cases, the serving size may be half a package but be misinterpreted as a complete package, or a consumer might take the serving size as a dietary recommendation rather than a description [18]. Some people disregard food labels entirely. Possible reasons include lack of time, disinterest, and habitual purchases [1, 19]. Others cited the belief that food labels are inaccurate and may contain false information that financially benefits companies as a barrier to reading food labels [20]. Food purchasing behaviors depend on the person's background in reading the food label and understanding whether the product is healthy or unhealthy. Educating people on how to use these labels could be a cost-effective, proactive method for the community to avoid buying unhealthy food [1, 4].

This study is aimed at investigating Zayed University students' knowledge, attitudes, and practices (KAPs) regarding food labels and to identify key predictors of food label use through logistic regression analysis. This age group is an interesting area of study as this tends to be a period when people begin forming their lifelong habits [21].

## 2. Methods

### 2.1. Ethical Approval and Clearance

This study was approved by the Research Ethics Committee at Zayed University, UAE (ZU22_088_F). All study participants provided informed consent prior to beginning the online questionnaire.

### 2.2. Study Design

A quantitative cross-sectional study was conducted in the UAE between January and May 2023 among students, predominantly female, aged 18 and above, from Zayed University in Dubai and Abu Dhabi campuses. A structured, self-designed online questionnaire in Arabic and English through Google Forms was distributed through WhatsApp and emails.

Zayed Unversity is a govermental university in the UAE, predominantly serving Emirati female students. The university's population aligns with national higher education trends, where women constitute a significant proportion of enrolled students due to active governmental encouragement of female education.

### 2.3. Sampling

A total of 1153 students were enrolled using snowball sampling. This method was chosen due to the difficulty of obtaining a comprehensive list of university students willing to participate in the study. Snowball sampling allowed us to efficiently reach a broad and diverse student population while maintaining a voluntary participation approach. However, we acknowledge that this method may introduce selection bias, as students who are more engaged in health-related discussions might be overrepresented. To mitigate this, we encouraged referrals from various academic disciplines and year groups to improve the diversity of the sample. Participants were provided information about the study and its purpose before answering the questionnaire and signed the consent form before participation; only approved participants were eligible to participate. The inclusion criteria were any student studying in Zayed University aged 18 and above. Students aged below 18 and those who did not give adequate answers were excluded. The participants had the right to withdraw from the study at any stage.

### 2.4. Data Collection

The data was collected using an online survey following a previously validated questionnaire while also increasing and modifying some questions and sections to ensure that the study's goal was reached [22, 23]. Through the questionnaire, we measured how much Zayed University students know about food labels and whether they use them personally. Additionally, it assessed participants' concerns about the expiry date and whether the product's price influences their reading of food labels. The survey included 27 questions. Considering the students' possible preference for one language over the other, the survey was written in English and then translated into Arabic. To ensure that the questions were clear, understandable, and error-free, the researcher reviewed them, and a pilot study of 20 participants from the College of Natural and Health Sciences was conducted to test the questionnaire. The pilot study was not included in the final collection. The questions were divided into three sections. The first section collected demographic information; it consisted of nine questions about age, gender, nationality, the emirate they live in, residential area, marital status, employment status, college, and year of study. The second section was about medical history and chronic diseases; it included only one question. The last section was about knowledge of food labeling, attitudes, and practices. It included 17 questions.

### 2.5. Statistical Analysis

The collected data was coded, entered, and analyzed using the Statistical Package for the Social Sciences (SPSS) version 26 (IBM, Armonk, New York, United States). Statistical tests with *p* values < 0.05 were considered statistically significant. Descriptive statistics were computed to summarize the questionnaire items, providing an overview of participants' knowledge and attitudes. Chi-square tests were employed to determine the statistical significance of the percentage differences observed between variables. Pearson correlation was used to examine the relationships between different variables, while binary logistic regression was applied to determine significant predictors of food label use.

Binary logistic regression was performed to determine predictors of food label usage. The dependent variable was whether students reported checking food labels (yes/no). Independent variables included demographic factors (age, sex, marital status, and employment status), shopping behaviors, and attitudes toward food labels. The model's assumptions were assessed for validity, and odds ratios (OR) with confidence intervals (CIs) were reported to measure the strength of associations.

## 3. Results

### 3.1. Descriptive Statistics

The demographic analysis revealed a predominantly female participant group.  Table 1 shows the study participants' demographic characteristics, consisting mainly of females (93.4%) with a mean age of 21.7 years (SD = 3.9). The majority fell within the 18–20 (44.0%) and 21–24 (42.2%) age categories, while a smaller proportion (13.8%) were aged 24 and above. Most participants were UAE nationals (94.8%), primarily residing in Dubai (59.6%), Abu Dhabi (15.8%), and Sharjah (13.6%). Urban areas were the predominant residential setting (81.7%) compared to rural areas (18.3%). Singles formed the majority in terms of participants (82.4%), followed by married (15.7%), divorced (1.3%), widowed (0.3%), and separated (0.3%) individuals. Most participants (65.8%) were unemployed, while 17.3% were full-time employed, and 8.1% were part-time employed or self-employed (8.8%). The natural and health sciences college had the highest representation (29.6%), followed by business (20.3%) and humanities and social sciences (12.3%). Fourth-year students comprised the largest group (33.4%), followed by third-year students (24.8%), and then second-year students (18.8%). A minority reported having chronic disease (17.3%), while the majority did not (82.7%).

The distribution of participants' knowledge about food labeling across various characteristics was examined using chi-square tests (Table 2). Significant associations were found between age groups (*χ*^2^ = 5.27, *p* = 0.07) and knowledge, with the highest proportion of knowledge observed in the 18–20 age group (88.4%) followed by the 21–24 age group (83.6%). This finding suggests that younger students may have higher exposure to nutrition-related education or awareness campaigns. However, the lack of a strong significance (*p* = 0.07) indicates that other factors might be influencing knowledge levels beyond just age.

Marital status also showed a significant association (*χ*^2^ = 7.67, *p* = 0.10), with married participants having more knowledge (80.7%). This could be attributed to the fact that married individuals may take greater responsibility for grocery shopping and meal preparation, making them more inclined to read and understand food labels. However, as the *p* value is above the significance threshold, this association should be interpreted with caution.

There were no significant associations between gender (*χ*^2^ = 1.12, *p* = 0.29), frequency of going shopping (*χ*^2^ = 2.65, *p* = 0.45), or emirates of residence (*χ*^2^ = 6.83, *p* = 0.34) and knowledge about food labeling. However, a significant association was found between educational colleges (*χ*^2^ = 14.17, *p* = 0.05), with the natural and health sciences college having the highest proportion of knowledge (90.9%).

**Figure 1:**
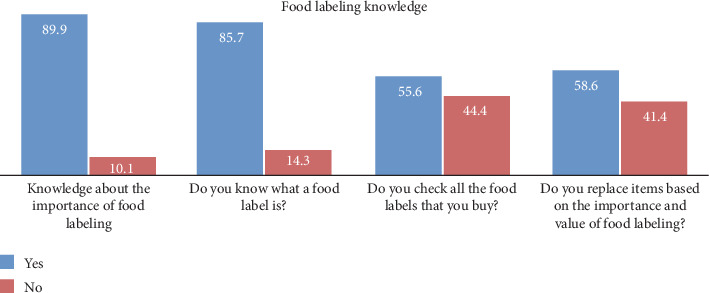
Knowledge and practice regarding food labeling (*N* = 1153).

Most participants indicated knowledge about the importance of food labeling, with 89.9% responding affirmatively and acknowledging its significance. A smaller proportion of respondents (10.1%) showed a lack of knowledge about the importance of food labeling. Regarding familiarity with food labels, 85.7% of participants reported knowing what a food label is, while 14.3% indicated a lack of knowledge in this area (Figure 1).

Table 3 summarizes the participants' distribution based on their practices related to food labeling. Over half (55.6%) of participants reported checking all the food labels for items they buy. Similarly, 58.6% of participants indicated that they replace food items based on the importance and value of food labeling. Regarding cost-based replacement, 61.8% (*n* = 712) of participants reported replacing food items based on cost. When asked about buying food items with no food labels, 41.9% of participants reported that they do so, while the majority (58.1%) reported not purchasing such items. The information participants looked for on food labels varied, with the highest percentages for calories (67.0%) and nutritional value (49.7%).

Regarding checking expiry dates, 85.8% of participants reported checking the expiry dates of every food item they buy, while 14.2% indicated not practicing this behavior. Additionally, only a small proportion (16.5%) reported buying expired items if available at low cost or for free, while the majority (83.5%) declared not engaging in such practice. Furthermore, most participants (58.1%) reported that low prices attract them to buy food items. In terms of preferring branded food items, the majority (74.0%) of the participants expressed a preference for branded items.

Similar to the analysis of knowledge about food labels, we compared attitudes toward food labeling among participants with and without chronic diseases.  Table 4 shows a significant association between attitudes toward the importance of food labeling and the presence of chronic diseases among the study participants (*χ*^2^ = 4.49, *p* = 0.034). This result suggests that individuals with chronic diseases are more likely to recognize the importance of food labeling compared to those without chronic diseases.

The results also showed a statistically significant relationship between the year of study and knowledge about food labels (*χ*^2^ = 12.245, *p* = 0.016). The chi-square test indicates that fourth-year students had the highest percentage of knowledge about food labels (87.8%), while first-year students had the lowest (75.8%). This suggests that knowledge about food labels tends to increase as students advance in their academic years.

### 3.2. Pearson Correlation

We computed the Pearson correlation between knowledge, attitude toward food labels, and behavior patterns concerning food labeling among different demographics. The results are shown in Tables 5 and 6.  Table 5 shows a moderately positive correlation between knowledge and attitude (0.265, *p* < 0.01), suggesting that as knowledge about food labeling increases, attitudes toward the importance of labeling also become more positive. This finding indicates that awareness often drives behavioral change. However, the moderate strength of the correlation suggests that knowledge alone is not sufficient to guarantee a strong positive attitude—external factors such as trust in food labels, accessibility of labeled products, or social influences might also play a role.

Furthermore, attitude toward food labeling shows a positive correlation with the practice of checking the expiration date (0.184), indicating that those with a more favorable view of food labeling are more diligent in checking expiration dates on food products.

Table 6 shows correlations between different perceptions and beliefs about food labeling. We observed a negative correlation between attitudes toward food labeling and perceptions toward food labels, which indicates that individuals with a positive attitude toward food labeling tend to be more skeptical about the source's trustworthiness and the various functionalities that labels are presumed to serve. Conversely, trustworthy source has strong positive correlations with the other perceptions about food label variables such as help health (0.406, *p* < 0.01), time reading (0.387, *p* < 0.01), regulate calories (0.335, *p* < 0.01), and easy label (0.396, *p* < 0.01), which indicates that those who find food labels to be a reliable source of information are also more likely to believe that labels can help with health management, take the time to read them, and find them effective for calorie regulation and easy to use.

### 3.3. Regression Analysis

Logistic regression analysis was conducted to identify key predictors of food label usage (dependent variable: checking food labels—yes/no). Independent variables included demographic factors (age, gender, marital status, and employment status) and shopping behaviors. This analysis helps determine which factors influence whether students actively engage with food labels (Table 7).

The model's intercept was statistically significant (*p* = 0.002), suggesting that the model does have a predictive value, although it does not come from these predictors. However, none of these demographic variables significantly predicted knowledge about food labeling, which is surprising given that prior literature often finds age and education level to be strong predictors. This could be due to the homogeneity of the sample, as most participants were university students of similar age and background.

Nonetheless, when considering attitudes toward the importance of food labeling, marital status and employment status emerged as significant predictors, with *p* values of 0.053 and < 0.001, respectively. Although the *p* values for marital status are slightly above the standard threshold of 0.05, it is close enough to warrant consideration.

The odds ratio for marital status (1.877) suggests that married individuals are 1.877 times more likely to perceive food labeling as necessary. At the same time, those with different employment statuses have a decreased likelihood of perceiving food labeling as necessary, with an odds ratio of 0.443. Regarding “checking and reading” food label variables, employment status was a significant predictor (*p* = 0.019), but age, gender, and marital status were not significant predictors. The odds ratio of employment status (1.370) indicates that the likelihood of checking and reading food labels varies based on employment status, with those being employed full-time or part-time being 1.370 times more likely to check and read food labels compared to other employment statuses.

This finding highlights the potential impact of life responsibilities on attitudes toward food labeling. Married individuals and employed participants may have more exposure to practical decision-making related to food purchases, reinforcing the relevance of food labels in their daily lives. The strong significance of employment status (*p* < 0.001) suggests that financial independence may also play a role in label-checking behavior, possibly because employed individuals have more control over their food choices.

## 4. Discussion

This study investigated the KAPs of university students regarding the use of nutritional information and food labels. The findings indicate a generally high level of awareness, with 89.9% of participants acknowledging the importance of food labeling and 85.7% reporting familiarity with food labels. These figures are notably higher than those reported in similar studies, where food label awareness ranged from moderate to low [24–27]. This contrasts with research from Saudi Arabia, where only 50%–60% of university students reported reading labels [24, 25]. The higher rates observed in our study could be attributed to the increased promotion of nutrition education programs and the strong presence of social media campaigns advocating for healthier choices.

### 4.1. Attitudes Toward Food Labels

Despite high awareness levels, actual food label utilization remains inconsistent. While 55.6% of participants reported checking labels regularly, a substantial proportion (44.4%) did not, similar to findings from studies in the United States and Europe, where many consumers fail to translate awareness into regular use [10, 28–31]. The most checked information on food labels included calories (67.0%) and nutritional value (49.7%), suggesting that students are concerned about weight management and health. However, this contrasts with studies from the UAE, Sudan, and Saudi Arabia, where consumers prioritized expiration dates over nutritional details [12, 22, 32]. This highlights a shift in awareness, where younger populations are more engaged with calorie monitoring than previous generations.

Interestingly, 41.8% of students considered food labels a trustworthy source, while 41.0% expressed some skepticism. This contradicts prior studies that found a high level of distrust in food labeling, particularly in regions with weaker regulatory enforcement [20]. The higher trust levels observed in our study could indicate growing confidence in food regulations within the UAE, possibly influenced by government-led initiatives promoting transparency in food labeling [33].

### 4.2. Impact of Chronic Disease Status on Label Use

A significant association was found between attitudes toward food labels and chronic disease status (*χ*^2^ = 4.49, *p* = 0.034). Students with chronic illnesses were more likely to value food labels, aligning with studies in South Korea and the United States, where patients managing diabetes, hypertension, or obesity were more likely to use nutritional labels [34, 35]. However, this contrasts with findings from studies in India and Turkey, where chronic disease patients were often unaware of food labeling regulations [33, 35]. Our study suggests that health education programs targeting chronic disease patients in the UAE may be more effective than in other regions.

### 4.3. Behavioral Trends and Influencing Factors

The price of food remained a dominant factor in purchasing decisions, with 61.8% of students replacing food items based on cost. This supports previous findings that financial constraints significantly influence food choices, particularly among college students and low-income populations [22, 36–38]. Additionally, 58.1% of students admitted being influenced by low prices, reinforcing studies from Malaysia and Australia, which found that financial barriers often override health considerations in food selection [38, 39]. This suggests that awareness alone is insufficient for promoting healthier food choices and calls for policy interventions that make healthier food options more affordable.

### 4.4. Demographic Predictors of Food Label Use

Regression analysis showed that marital status and employment status were the strongest predictors of attitudes toward food labeling (*p* = 0.053 and *p* < 0.001, respectively). Married students were nearly twice as likely (OR = 1.877) to perceive food labeling as important, likely due to their responsibility in household food purchasing. This is consistent with findings from studies in Canada and the United Kingdom, where married consumers demonstrated greater label engagement [40, 41].

Similarly, employed students were 1.370 times more likely to check food labels, which aligns with research from some countries like Canada and Brazil, where higher income groups showed greater label use [39, 42, 43]. This suggests that financial independence plays a key role in food label engagement. However, unlike previous studies that reported age and gender as strong predictors of food label use [41, 44], these factors were not significant in our study. This may be due to the homogeneous nature of the sample, where most participants were young, female university students. These results suggest that targeted education campaigns should consider life-stage factors to enhance food label literacy among students.

### 4.5. Policy and Education Implications

Our findings suggest that nutrition education programs should be expanded beyond awareness campaigns to address behavioral barriers, particularly cost-related obstacles to healthy eating. Public health initiatives should target students with chronic diseases, given their higher likelihood of benefiting from food label use. Additionally, policymakers could explore incentives for healthier food choices, such as subsidized pricing for nutritious products and mandatory clearer front-of-pack labeling.

These findings highlight that high awareness of food labels does not always translate into consistent use, with price and convenience still dominating food selection. However, the strong associations between chronic disease status, employment, and label use suggest key groups that would benefit from targeted nutrition education. Moving forward, public health strategies should focus on making healthier food choices more accessible and affordable while reinforcing trust in food labeling as a reliable source of information.

### 4.6. Limitations

This study has some limitations that should be acknowledged. First, the sample was limited to Zayed University students, making it difficult to generalize findings to other universities or broader populations. Future research should include a more diverse sample across different institutions and demographics to provide a more representative understanding of food labeling knowledge and behaviors.

Second, this study did not examine how misinformation, skepticism, or negative perceptions about food labels affect consumer behavior. Prior research suggests that some individuals distrust food labels due to perceived inaccuracy or industry bias, which can significantly impact their usage. Addressing this gap could provide deeper insights into barriers to effective label use.

Third, the study relied on self-reported data collected through an online survey, which may introduce response bias or misinterpretation of questions. Without direct researcher clarification, participants may have misunderstood certain terms or provided socially desirable responses rather than reflecting their true practices. Future studies should consider mixed-method approaches, such as in-depth interviews or observational studies, to validate self-reported behaviors. Moreover, the study did not explore the long-term impact of food label literacy on actual dietary habits. While many students reported checking food labels, it is unclear whether this translates into healthier eating patterns over time. Longitudinal studies could assess whether food label awareness leads to sustained improvements in diet and health outcomes.

## 5. Conclusion

Our findings highlight the potential of food labels as an effective tool for improving nutritional awareness and decision-making among university students. The high levels of knowledge and positive attitudes toward food labeling suggest that students recognize its value, but actual usage remains inconsistent. This highlights the need for targeted education programs that go beyond awareness and emphasize practical strategies for effectively interpreting and applying food label information.

Given the strong association between chronic disease status and food label usage, integrating food label literacy into health education programs could be particularly beneficial for students with specific dietary needs or health conditions. Additionally, addressing price-related barriers to healthy food choices through policy interventions or university-led nutrition initiatives could further support students in making informed decisions.

Moving forward, collaborations between educational institutions, health professionals, and policymakers can help enhance food label literacy, reduce misinformation, and encourage healthier food choices. By making nutritional information more accessible and understandable, food labels can play a crucial role in shaping long-term dietary habits and preventing diet-related diseases.

## Figures and Tables

**Table 1:** Participant demographic characteristics.

**Characteristic**	**Category**	**Frequency**	**%**
Age group	18–20	507	44.0
	21–24	487	42.2
	24+	159	13.8

Gender	Female	1077	93.4
	Male	76	6.6

Nationality	UAE national	1093	94.8
	Non-UAE national	60	5.2

Emirates	Abu Dhabi	182	15.8
	Dubai	687	59.6
	Sharjah	157	13.6
	Ras Al Khaimah	29	2.5
	Ajman	62	5.4
	Umm Al Quwain	21	1.8
	Fujairah	15	1.3

Residential area	Urban	942	81.7
	Rural	211	18.3

Marital status	Married	181	15.7
	Single	949	82.4
	Divorced	15	1.3
	Widowed	4	0.3
	Separated	4	0.3

Employment status	Employed full-time	200	17.3
	Employed part-time	93	8.1
	Self-employed	101	8.8
	Unemployed	759	65.8

Education	Arts and creative enterprises	100	8.7
	Natural and health sciences	342	29.6
	Humanities and social sciences	142	12.3
	Education	77	6.7
	Business	235	20.3
	Communication and media sciences	115	10.0
	Technological innovation	101	8.8
	Interdisciplinary studies	41	3.6

Study year	1st year	124	10.8
	2nd year	217	18.8
	3rd year	286	24.8
	4th year	385	33.4
	5th+ year	141	12.2

Chronic disease	Yes	199	17.3
	No	954	82.7

**Table 2:** Distribution of respondents according to knowledge about food labeling.

**Characteristics**		**Knowledge about food labeling**			**Total**	**Chi-square, ** **p** ** value**
			**No**	**Yes**		
Age groups	18–20	*N*	448	59	507	5.27, 0.07
		%	88.4%	11.6%	100%	
	21–24	N	407	80	487	
		%	83.6%	16.4%	100%	
	24+	*N*	133	26	159	
		%	83.6%	16.4%	100%	

Marital status	Married	*N*	146	35	181	7.67, 0.10
		%	80.7%	19.3%	100%	
	Single	*N*	820	129	949	
		%	86.4%	13.4%	100%	
	Divorced	*N*	15	0	15	
		%	100%	0%	100%	
	Widowed	*N*	3	1	4	
		%	75%	25%	100%	
	Separated	*N*	4	0	4	
		%	100%	0%	100%	

Gender	Female	*N*	926	151	1077	1.12, 0.29
		%	86.0%	14.0%	100%	
	Male	*N*	62	14	76	
		%	81.6%	18.4%	100%	

Education	Arts and creative enterprises	*N*	84	16	100	14.17, 0.05
		%	84%	16%	100%	
	Natural and health sciences	*N*	311	31	342	
		%	90.9%	9.1%	100%	
	Humanities and social sciences	*N*	123	19	142	
		%	86.6%	13.4%	100%	
	Education	*N*	63	14	77	
		%	81.8%	18.2%	100%	
	Business	*N*	190	45	235	
		%	80.9%	19.1%	100%	
	Communication and media sciences	*N*	96	19	115	
		%	83.5%	16.5%	100%	
	Technological innovation	*N*	87	14	101	
		%	86.1%	13.9%	100%	
	Interdisciplinary studies	*N*	34	7	41	
		%	82.9%	17.1%	100%	

Shopping frequency	Once a week	*N*	220	36	256	2.65, 0.45
		%	85.9%	14.1%	100%	
	Twice a week	*N*	138	29	167	
		%	82.6%	17.4%	100%	
	Monthly	*N*	333	47	380	
		%	87.6%	12.4%	100%	
	Occasionally	*N*	297	53	350	
		%	84.9%	15.1%	100%	

Year of study	1st year	*N*	94	30	124	12.25, 0.02
		%	75.8%	24.2%	100%	
	2nd year	*N*	188	29	217	
		%	86.6%	13.4%	100%	
	3rd year	*N*	250	36	286	
		%	87.4%	12.6%	100%	
	4th year	*N*	338	47	385	
		%	87.8%	12.2%	100%	
	5th+ year	*N*	117	24	141	
		%	83.0%	17.0%	100%	

Emirates	Abu Dhabi	*N*	154	28	182	6.83, 0.34
		%	84.6%	15.4%	100%	
	Dubai	*N*	589	98	687	
		%	85.7%	14.3%	100%	
	Sharjah	*N*	139	18	157	
		%	88.5%	11.5%	100%	
	Ras Al Khaimah	*N*	22	7	29	
		%	75.9%	24.1%	100%	
	Ajman	*N*	56	6	62	
		%	90.3%	9.7%	100%	
	Umm Al Quwain	*N*	17	4	21	
		%	81.0%	19.0%	100%	
	Fujairah	*N*	11	4	15	
		%	73.3%	26.7%	100%	

**Table 3:** Distribution of subjects based on practice in food labeling.

**Variables**	**Response**	**N**	**%**
*Food labeling*			
Do you check all the food labels that you buy?	Yes	641	55.6
	No	512	44.4

Do you replace items based on the importance and value of food labeling?	Yes	675	58.6
	No	478	41.4

Do you replace food items based on cost?	Yes	712	61.8
	No	441	38.2

Do you buy food items with no food labels?	Yes	483	41.9
	No	670	58.1

What do you see on a food label?	Calories	772	67.0
	Place of manufacturing	542	47.0
	Nutritional value	573	49.7
	Production and expiry date	821	71.2
	Claims about nutrition	520	45.1
	Claims about health	460	39.9
	Ingredient list	611	53.0
	All the above	313	27.1
	I do not read the food label	100	8.7

What do you see on a food label?	Calories	73	6.3
	Expiry date	55	4.8
	Place of manufacturing	9	0.8
	Calories + nutritional value	2	0.2
	Calories + nutritional value + expiry date	24	2.1
	Nutritional value + expiry date	5	0.4

*Expiry dates*			
Do you check the expiry dates of every food you buy?	Yes	989	85.8
	No	164	14.2

Do you buy anything expired if available at low cost or free?	Yes	190	16.5
	No	963	83.5

*Characteristics for buying food items*			
Do low prices attract you to buy food items?	Yes	670	58.1
	No	483	41.9

Do you prefer to buy branded food items?	Yes	853	74.0
	No	300	26.0

Total		1153	100.0

**Table 4:** Distribution of respondents according to attitude toward food labels.

**Characteristics**		**Attitude toward food labeling**			**Total**	**Chi-square, ** **p** ** value**
			**No**	**Yes**		
Chronic disease	Yes	*N*	154	54	199	4.49, 0.034
		%	77.4%	22.6%	100%	
	No	*N*	882	72	954	
		%	92.5%	7.5%	100%	

**Table 5:** Pearson correlations between knowledge, attitudes, and demographic variables.

	**Knowledge**	**Attitude**	**Shopping frequency**	**Food replacement**	**No labels**	**Expiry date**	**Age**	**Gender**	**Marital status**	**Employment status**	**Education**	**Study year**	**Chronic disease**
Knowledge	1	0.265⁣^∗∗^	−0.002	0.154⁣^∗∗^	−0.050	0.153⁣^∗∗^	0.063⁣^∗^	0.031	−0.067⁣^∗^	−0.076⁣^∗∗^	0.064⁣^∗^	−0.061⁣^∗^	−0.062⁣^∗^
Attitude		1	−0.043	0.160⁣^∗∗^	−0.041	0.184⁣^∗∗^	0.024	0.015	0.076⁣^∗∗^	−0.078⁣^∗∗^	0.027	−0.035	−0.189⁣^∗∗^
Shopping frequency			1	0.049	0.044	0.008	−0.009	−0.023	0.082⁣^∗∗^	0.092⁣^∗∗^	−0.052	0.070⁣^∗^	0.140⁣^∗∗^
Food replacement				1	0.047	0.071⁣^∗^	−0.055	−0.004	0.109⁣^∗∗^	0.072⁣^∗^	−0.016	−0.023	0.035
No labels					1	−0.052	−0.001	−0.079⁣^∗∗^	0.039	0.125⁣^∗∗^	0.018	−0.017	0.110⁣^∗∗^
Expiry date						1	0.003	0.012	0.043	−0.044	−0.031	−0.009	−0.110⁣^∗∗^
Age							1	0.083⁣^∗∗^	−0.311⁣^∗∗^	−0.256⁣^∗∗^	0.088⁣^∗∗^	0.365⁣^∗∗^	−0.091⁣^∗∗^
Gender								1	−0.026	−0.237⁣^∗∗^	0.069⁣^∗^	−0.040	−0.064⁣^∗^
Marital status									1	0.114⁣^∗∗^	−0.068⁣^∗^	−0.043	−0.001
Employment status										1	−0.093⁣^∗∗^	−0.069⁣^∗^	0.201⁣^∗∗^
Education											1	−0.097⁣^∗∗^	0.065⁣^∗^
Study year												1	0.007
Chronic disease													1

^**^Correlation is significant at the 0.01 level.

^*^Correlation is significant at the 0.05 level.

**Table 6:** Pearson correlation between knowledge, attitudes, and behavior patterns concerning food labeling.

	**Attitude**	**Trustworthy source**	**Help health**	**Time reading**	**Regulate calories**	**Easy label**
Attitude	1	−0.180⁣^∗∗^	−0.246⁣^∗∗^	−0.236⁣^∗∗^	−0.228⁣^∗∗^	−0.162⁣^∗∗^
Trustworthy source		1	0.406⁣^∗∗^	0.387⁣^∗∗^	0.335⁣^∗∗^	0.396⁣^∗∗^
Help health			1	0.475⁣^∗∗^	0.352⁣^∗∗^	0.328⁣^∗∗^
Regulate calories				1	0.497⁣^∗∗^	0.363⁣^∗∗^
Time reading					1	0.409⁣^∗∗^
Easy label						1

^**^Correlation is significant at the 0.01 level.

^*^Correlation is significant at the 0.05 level.

**Table 7:** Logistic regression for the knowledge, attitude, and reading food label variables.

**Variables knowledge, attitude, and reading food labels**	**Coefficient** **β**	**Standard error (SE)**	**Wald**	**df**	**p** ** value**	**Odds ratio Exp(** **β** **)**	**95% CI for Exp(** **β** **)**
Constant	−1.854	0.595	9.715	1	0.002	0.157	
	−2.598	0.780	11.083	1	< 0.001	0.074	
	−0.862	0.480	3.222	1	0.073	0.422	

Age	0.021	0.021	1.002	1	0.317	1.021	(0.98, 1.06)
	0.016	0.027	0.366	1	0.545	1.017	(0.96,1.07)
	0.006	0.017	0.143	1	0.705	1.006	(0.97, 1.04)

Gender	0.124	0.320	0.149	1	0.699	1.132	(0.60, 2.12)
	-0.081	0.380	0.046	1	0.831	0.922	(0.44, 1.94)
	-0.222	0.255	0.760	1	0.383	0.801	(0.49, 1.32)

Marital status	-0.254	0.236	1.163	1	0.281	0.775	(0.49, 1.23)
	0.630	0.325	3.744	1	0.053	1.877	(0.99, 3.55)
	0.356	0.186	3.667	1	0.55	1.428	(0.99, 2.06)

Employment status	−0.313	0.182	2.956	1	0.086	0.731	(0.51, 1.05)
	−0.814	0.208	15.267	1	< 0.001	0.443	(0.30, 0.67)
	0.315	0.134	5.498	1	0.019	1.370	(1.05, 1.78)

## Data Availability

The data that support the findings of this study are available on request from the corresponding author. The data are not publicly available due to privacy or ethical restrictions.
